# Antemortem detection of Parkinson’s disease pathology in peripheral biopsies using artificial intelligence

**DOI:** 10.1186/s40478-022-01318-7

**Published:** 2022-02-14

**Authors:** Maxim Signaevsky, Bahram Marami, Marcel Prastawa, Nabil Tabish, Megan A. Iida, Xiang Fu Zhang, Mary Sawyer, Israel Duran, Daniel G. Koenigsberg, Clare H. Bryce, Lana M. Chahine, Brit Mollenhauer, Sherri Mosovsky, Lindsey Riley, Kuldip D. Dave, Jamie Eberling, Chris S. Coffey, Charles H. Adler, Geidy E. Serrano, Charles L. White, John Koll, Gerardo Fernandez, Jack Zeineh, Carlos Cordon-Cardo, Thomas G. Beach, John F. Crary

**Affiliations:** 1 Department of Pathology, Icahn School of Medicine at Mount Sinai, 1 Gustave L. Levy Place, Box 1194, New York, NY 10029 USA; 2 Center for Computational and Systems Pathology, Icahn School of Medicine at Mount Sinai, New York, NY USA; 3 Neuropathology Brain Bank and Research Core, Icahn School of Medicine at Mount Sinai, New York, NY USA; 4 Ronald M. Loeb Center for Alzheimer’s Disease, Friedman Brain Institute, Icahn School of Medicine at Mount Sinai, New York, NY USA; 5 Nash Family Department of Neuroscience, Icahn School of Medicine at Mount Sinai, New York, NY USA; 6 Department of Neurology, University of Pittsburgh, Pittsburgh, PA USA; 7 Center of Parkinsonism and Movement Disorders Paracelsus, Elena Klinik Kassel and University Medical Center Göttingen, Göttingen, Germany; 8 The Michael J. Fox Foundation for Parkinson’s Research, New York, NY USA; 9 University of Iowa, Iowa City, IA USA; 10 Department of Neurology, Mayo Clinic College of Medicine, Scottsdale, AZ USA; 11 Civin Laboratory for Neuropathology, Banner Sun Health Research Institute, Sun City, AZ USA; 12 Neuropathology Laboratory, Department of Pathology, UT Southwestern Medical Center, Dallas, TX USA; 13 Department of Artificial Intelligence and Human Health, Icahn School of Medicine at Mount Sinai, New York, NY USA

**Keywords:** Artificial intelligence, Machine learning, Deep learning, Convolutional neural network, Whole slide image, Parkinson’s disease, Synucleinopathy, Peripheral biopsy, Submandibular gland

## Abstract

The diagnosis of Parkinson’s disease (PD) is challenging at all stages due to variable symptomatology, comorbidities, and mimicking conditions. Postmortem assessment remains the gold standard for a definitive diagnosis. While it is well recognized that PD manifests pathologically in the central nervous system with aggregation of α-synuclein as Lewy bodies and neurites, similar Lewy-type synucleinopathy (LTS) is additionally found in the peripheral nervous system that may be useful as an antemortem biomarker. We have previously found that detection of LTS in submandibular gland (SMG) biopsies is sensitive and specific for advanced PD; however, the sensitivity is suboptimal especially for early-stage disease. Further, visual microscopic assessment of biopsies by a neuropathologist to identify LTS is impractical for large-scale adoption. Here, we trained and validated a convolutional neural network (CNN) for detection of LTS on 283 digital whole slide images (WSI) from 95 unique SMG biopsies. A total of 8,450 LTS and 35,066 background objects were annotated following an inter-rater reliability study with Fleiss *Kappa* = 0.72. We used transfer learning to train a CNN model to classify image patches (151 × 151 pixels at 20× magnification) with and without the presence of LTS objects. The trained CNN model showed the following performance on image patches: sensitivity: 0.99, specificity: 0.99, precision: 0.81, accuracy: 0.99, and F-1 score: 0.89. We further tested the trained network on 1230 naïve WSI from the same cohort of research subjects comprising 42 PD patients and 14 controls. Logistic regression models trained on features engineered from the CNN predictions on the WSI resulted in sensitivity: 0.71, specificity: 0.65, precision: 0.86, accuracy: 0.69, and F-1 score: 0.76 in predicting clinical PD status, and 0.64 accuracy in predicting PD stage, outperforming expert neuropathologist LTS density scoring in terms of sensitivity but not specificity. These findings demonstrate the practical utility of a CNN detector in screening for LTS, which can translate into a computational tool to facilitate the antemortem tissue-based diagnosis of PD in clinical settings.

**Supplementary Information:**

The online version contains supplementary material available at 10.1186/s40478-022-01318-7.

## Introduction

Parkinson’s disease (PD), dementia with Lewy bodies, and multiple system atrophy, which are all characterized histologically by intracellular aggregates of misprocessed α-synuclein, are the most common among the spectrum of synucleinopathies, and are the second most common neurodegenerative conditions after Alzheimer disease (AD), with PD being the most common in this group [23]. It is widely accepted that accurate and early diagnosis of PD is needed for meaningful therapeutic approaches for disease modification [30]. Postmortem assessment of brain tissue by an expert neuropathologist remains the only option and gold standard for a definitive diagnosis of PD [39]. The antemortem diagnosis is challenging due to a variable clinical presentation and several mimicking conditions, exemplified most commonly by dementia with Lewy bodies, multiple system atrophy, and progressive supranuclear palsy, obscuring the clinical picture, and delaying treatment [2, 44]. Many clinical trials in PD have failed to identify disease-modifying therapies [8]. Robust and accurate PD biomarkers are crucial to enable early diagnosis, test for target engagement, and serve as surrogate measures of disease in clinical trials.

While PD is diagnosed based on identification of misprocessed α-synuclein in Lewy bodies and Lewy neurites in the central nervous system (CNS), these pathological changes can be also found in peripheral nerves [1, 4, 7, 9, 11, 36, 42, 46]. The reports on occurrence of pathologic α-synuclein in peripheral tissue in PD are often conflicting [3, 4, 7, 15, 21, 22, 28, 32, 40, 42, 43, 50], which could be attributed to a range of methodological factors including specimen acquisition/processing, α-synuclein staining methods, uncertainty of clinical diagnosis, neuropathologist expertise, and blinding [32]. The diagnostic and prognostic value of peripheral biopsy in PD has gained recognition, including in the gastrointestinal tract, salivary glands, olfactory mucosa, and skin [36]. The submandibular gland has particular potential due to the high density of nerves containing pathological α-synuclein as well as its accessibility to biopsy [5, 13, 37, 38, 46]. Lewy-type pathology in the submandibular gland has been detected and characterized in earlier PD stages [3, 15, 21, 50], and its high specificity and good sensitivity has been shown [13, 36]. It has also been observed through systemic peripheral sampling that Lewy-type pathology generally follows a rostro-caudal distribution [3, 43]. The highest densities of Lewy type α-synucleinopathy (LTS) are in the lower esophagus and submandibular gland and lowest in the colon and rectum [3, 10, 43]. More recently, there has been increasing interest in using skin biopsies as a screening and prognostication tool in PD but the density of synuclein pathology-containing nerves is much lower, reducing diagnostic sensitivity [7, 18–22]. Such assessment is subject to inter- and intra-observer variability and represents a laborious and time-consuming process, which limits its practical applications.

Artificial intelligence (AI) in the context of computer vision could be employed to the improve diagnostic utility of peripheral biopsies in PD. AI has been shown to be promising in cancer pathology in screening, detection, and predictive modeling [25]. Its potential in medical imaging of the central nervous system, such as magnetic resonance imaging (MRI) and positron emission tomography (PET), has been explored by many groups [6, 24, 29, 31, 33, 34, 41]. However, the application of AI to histological preparations in neuropathology to date is limited. Our group developed and published the first neurofibrillary tangle classifier applicable to Alzheimer disease (AD), primary age-related tauopathy (PART), and other primary tauopathies [47]. Wurts et al. [53] have presented a report on pathological histomorphological forms of tau in whole slide images (WSI) of AD brain samples. Similarly, Tang et al. [49] have demonstrated that neuritic amyloid plaques and cerebral amyloid angiopathy can be detected with a high degree of precision and recall. This has been validated by Vizcarra et al. [52] in a multicenter study. Together, these studies reinforce the assertion that computer vision and machine learning will have broad applications in the histological assessment of tissues from patients with neurodegenerative diseases.

Here, we applied deep-learning based classification to a collection of WSIs from the Systemic Synuclein Sampling Study (S4), a large multicenter study initiated with the goal of assessing key gaps in knowledge by comparing inter- and intra-individual total α-synuclein in central and peripheral fluid compartments (i.e., cerebrospinal fluid, blood, saliva), and the occurrence of immunohistochemically-defined α-synuclein pathology in three peripheral tissues (i.e., colon, skin, and submandibular gland) at different PD stages compared to controls [1, 11, 13, 14, 51]. Our previous results indicate that peripheral Lewy-type synucleinopathy (LTS) is present in early PD, suggesting its utility as a diagnostic and prognostic biomarker. The expert neuropathologist semi-quantitative assessment of LTS in peripheral biopsies was shown to have nearly perfect specificity in predicting clinical diagnosis and stages of PD; however, the sensitivity was moderate [11, 13]. We applied a trained convolutional neural network (CNN) to minimize barriers to wider application of LTS assessment for early diagnosis of PD. This approach provides robust and reliable quantitative measurements of an array of AI-based features representing LTS burden and distribution on the digitized peripheral biopsy WSI, with potential to be further used as diagnostic, prognostic, and monitoring markers of PD.

## Materials and methods

### Case materials

The Systemic Synuclein Sampling Study (S4) was a cross-sectional, observational six-site study. Methodology has previously been described, including cohort inclusion/exclusion criteria [13, 51]. Briefly, the study included 60 individuals with idiopathic Parkinson’s disease and 20 controls with specimens of submandibular gland (SMG), skin, colon, CSF, and blood. The PD group consisted of individuals with a clinical diagnosis of PD and abnormal dopamine transporter SPECT imaging, with either early (2 or less years duration, untreated with dopaminergic medication), moderate (2–5 years duration, without motor fluctuations), or advanced (more than 5 years duration with motor fluctuations) disease. Controls consisted of individuals with normal dopamine transporter imaging. Both groups had to be free of dementia and medical conditions that precluded study procedures. All participants underwent Movement Disorders Society Unified Parkinson’s Disease Rating Scale (MDS-UPDRS) assessment, as previously described (Table 1) [13, 27]. Cerebrospinal fluid (CSF) was obtained via lumbar puncture and total α-synuclein was measured using ELISA as described [14, 51]. Dopamine transporter SPECT scans were obtained and striatal specific binding ratio (SBR) was calculated as mean SBR for right and left putamen and caudate. Biopsies of SMG were obtained and stained as described [1, 11, 14, 51]. Briefly, up to 5 unilateral biopsies per participant were fixed in 10% formalin, embedded in paraffin and cut into 4 μm sections that were then mounted on glass slides [1]. Up to 13 slides from each paraffin block were then treated with a protease and stained with the 5C12 mouse monoclonal antibody. The 42 PD participants and 14 controls whose SMG biopsies yielded adequate tissue were included in the present analysis.

**Table 1:** Subject data

	Control	Parkinson disease, total	Parkinson disease, stage		
			Early	Moderate	Advanced
*n* (male/female)	14 (8/6)	42 (30/12)	15 (13/2)	13 (8/5)	14 (9/5)
Age (yr)*	62.5 ± 6.5	64.5 ± 9.2	63.8 ± 10.5	58.8 ± 6.6	70.5 ± 5.9
Disease duration (mo)	–	61.1 ± 64.4	10.5 ± 7.2	44.8 ± 17.8	130.4 ± 56.2
MDS-UPDRS*					
Part I	2.6 ± 2.4	8.0 ± 5.3	8.1 ± 6.0	7.1 ± 4.5	8.6 ± 5.7
Part II	0.1 ± 0.4	10.1 ± 6.4	8.9 ± 5.8	8.7 ± 4.0	12.7 ± 8.2
Part III	1.1 ± 2.7	26.1 ± 12.0	20.1 ± 9.9	27.1 ± 11.8	31.9 ± 12.1
Total	3.8 ± 4.0	44.0 ± 18.8	37.2 ± 17.7	42.9 ± 14.9	52.9 ± 21.1

*MDS-UPDRS* Movement Disorders Society Unified Parkinson Disease Rating Scale

*Mean ± SD

### Slide digitization and image management

The stained glass slides were digitalized at 20× magnification using Aperio scanners (Aperio, Leica Biosystems, Kassel, Germany) and the whole slide image (WSI) files were saved in.svs format. A total of 1513 WSIs were used in this study, with 283 used for the development of LTS detector (training and testing of the neural networks) and 1230 for the feature generation and the development of PD status/stage prediction models. The WSI were then converted into a GeoTIFF format. Images were stored on a HIPAA-compliant server behind the hospital firewall for interactive display and annotation over the intranet using the Precise Informatics Platform (PIP), developed by the Center for Computational and Systems Pathology at Mount Sinai (MP, JK, JZ, and GF), which allows for the management of thousands of images with pathologist annotations.

### Expert neuropathologist scoring

**Fig. 1:**
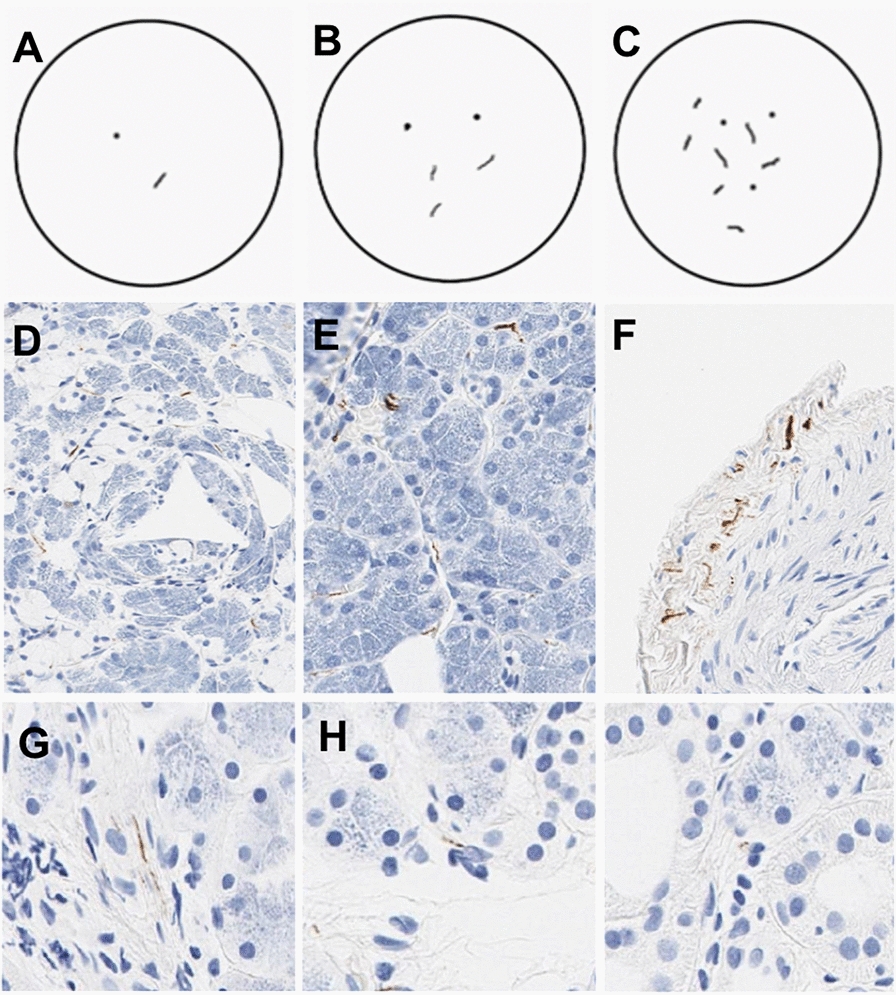
LTS object scoring and confidence ranking. Illustration of scoring density of sparse (**A**) moderate (**B**), and frequent (**C**). Submandibular gland biopsies immunohistochemically stained for α-synuclein with examples of sparse (**D**) moderate (**E**) and frequent (**F**). Examples of confidence ranking examples of definite (**G**), probable (**H**), and possible (**I**). LTS can be visualized and single or multiple immunopositive normal or misshapen axonal profiles in the submandibular gland parenchyma, nerve fascicles, or adjacent to blood vessels. *LTS* Lewy-type synucleinopathy

Digitized images were previously scored by three independent neuropathologists blinded to diagnostic group as described [1, 11, 13]. Briefly, each WSI was classified as positive or negative for α-synuclein pathology and assigned an LTS score ranging from 0 to 3 (Fig. 1), where 0 refers to being negative for α-synuclein, and scores 1–3 refer to scoring density of sparse (1) moderate (2), and frequent (3). A final determination regarding IHC positivity for α-synuclein was made for each slide based on consensus rating of at least 2/3 pathologists; each study subject was assigned derivative score metrics, reflecting cumulative α-synuclein positivity (Additional file 1: Table S2) [11, 13, 14].

### Pathological annotations

Ground truth annotations were generated using the Precise Informatics Platform collaborative web-based user interface. We delineated operational morphological definitions of the LTS based our previous studies (Fig. 1A–F) with modifications [1, 11]. Briefly, LTS was operationalized as an IHC α-synuclein-positive object, i.e. “foreground”, with a neurite-like morphology in an appropriate histological context (e.g., periacinar/gland parenchyma, perivascular, or within a nerve bundle). Given high histomorphological variability of LTS and their planes of section, each LTS object was assigned a confidence rank (i.e., definite, probable, and possible) reflecting an annotator’s confidence for further inclusion in weighted computational modeling (Fig. 1D–I). Objects showing IHC α-synuclein-positive linear neurites in an appropriate histological context were scored as “definite”. Single or multiple dot-like objects possibly representing a cross-section of α-synuclein-positive neurites were classified as “possible” reflecting the lower annotator’s confidence. The “probable” rank was used for intermediate confidence. Other α-synuclein immunopositive structures including granules/grains, macrophages, and artifacts were categorized as “background”. This approach increased the number of classifiable objects for training. To assess the annotated ground truth, we conducted annotator training and a concordance study to measure the inter-rater reliability, using a custom interface within the PIP platform, and compared them using a Fleiss’ kappa statistic and percentage agreement.

### Neural network training

**Fig. 2:**
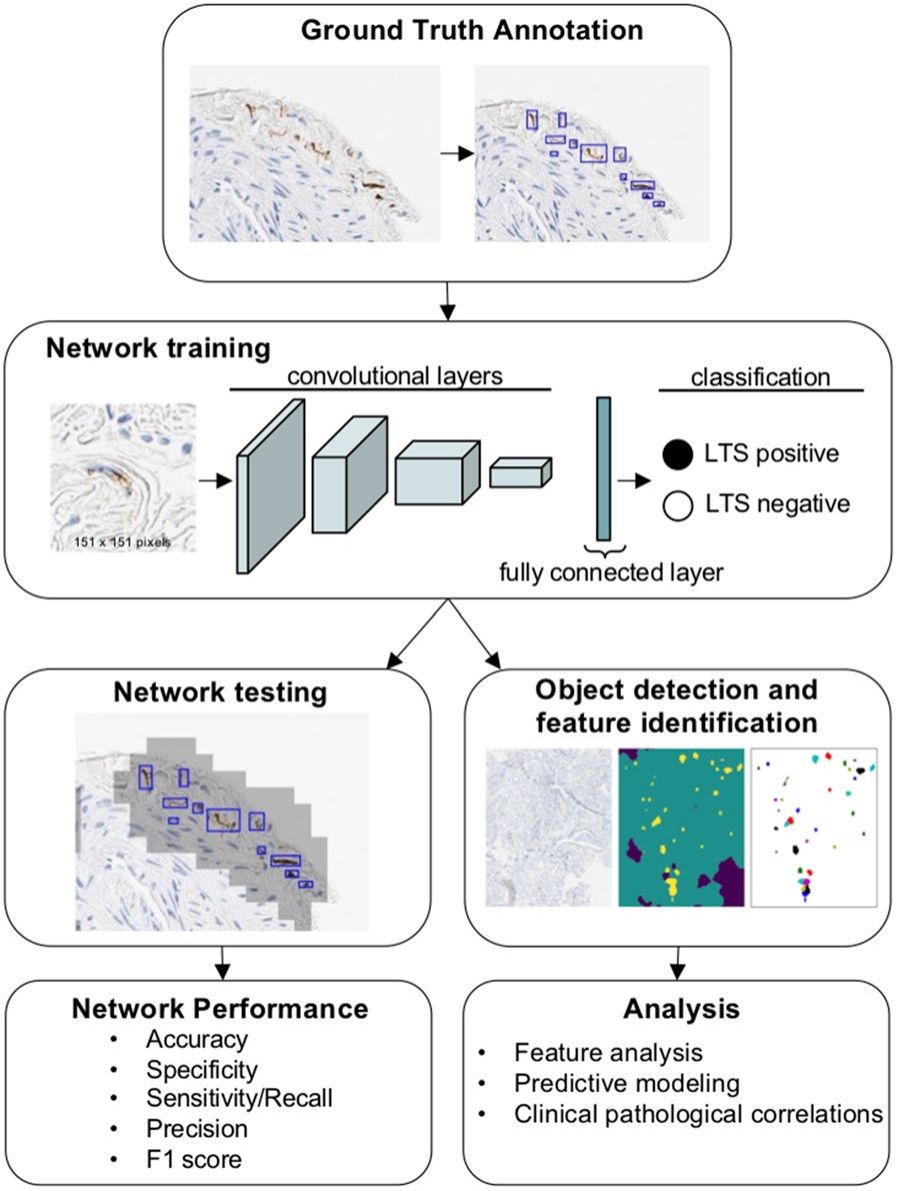
Schematic overview of data annotation and deep learning pipeline. LTS are annotated using WSI. The CNN was trained to classify image patches containing LTS from other image patches including tissue, artifacts and background. Different weights were used for the annotated objects while training using the cross entropy loss function for the final network. Image patches are extracted for network training that generates pixel-wise segmentations for LTS and background. Performance is determined using a separate novel set of images (test set) by comparing expert annotation with the trained network. The resulting trained network is further deployed on the naive WSI dataset for assessment of the predictive power of clinical outcomes. *LTS* Lewy-type synucleinopathy, *WSI* whole slide image, *CNN* convolutional neural network

We used a convolutional neural network (CNN) architecture (InceptionV4 [48]) to train the LTS detector (Fig. 2). The CNN was trained using a system with five NVIDIA Titan Xp GPUs and Intel(R) Xeon(R) CPU E5-2660 v4 @ 2.00 GHz. The convolutional layers in the CNN were initialized with imagenet [16] pre-trained weights. We also modified the first convolutional layer in the InceptionV4 architecture and set the stride equal to 1, which allowed us to use a smaller image size (151 × 151) than the original imagenet model (299 × 299). We used the PyTorch platform for training and creating the prediction masks. When training, various image augmentation methods were applied, including smoothing, sharpening, rotations, flips, jpeg compression, as well as contrast and brightness adjustments. An AdamW optimizer with a learning rate of 0.00001 and weight decay of 0.001 was used for training the final CNN detector [35]. The mini-batch size was 32 per GPU and the network was trained 80 epochs. Definite, probable and possible LTS patches were assigned loss weights equal to 5, 4, and 3, respectively. False positive patches from earlier rounds of training were given a loss weight equal to 2. Artifacts, background and patches with no LTS objects were given a loss weight equal to 1. Higher weights corresponded to larger misclassification penalty. After the training of the CNN detector, we created the prediction masks on the whole slide images by performing the inference on overlapping patches with 40 pixels strides.

### Derivation of image features

To assess the relationship between the detected Lewy-type synucleinopathy objects in the whole slide images and the disease status and progression, we used 60 different image features based on not only the total detected LTS burden, but also their spatial distribution and staining variations to identify patterns similar to what experts consider when producing their assessment score (Additional file 1: Table S3). The PreciseDx features describe the organization of the detected Lewy neurite objects in the whole slide. It includes the local relationship of each object to its neighbors, based on measures of the node degree property in a graph constructed from the objects. These measures provide summary descriptions on the patterns of Lewy neurites in tissue. It also includes descriptions on the separation of the detected objects, measuring the homogeneity of their distributions in tissue. The collection of features can characterize the whole slide in regards to Lewy neurites and we investigate their clinical utility with disease burden. Number of detected LTS patches and the fraction of the detected LTS patches within the overall tissue region represent the LTS load for each slide. To assess the stain variations within the detected LTS patches, we separated the IHC/DAB staining (brown) from the hematoxylin counterstaining (blue) using color deconvolution [45]. After color deconvolution, we computed the sum, mean, median and standard deviation of the hematoxylin and DAB within the detected LTS patches for each slide.

To identify the spatially connected and coherent regions and their distribution patterns, we used affinity propagation to find clusters of the detected LTS patches within the WSI [12, 26]. From the resulting clusters, measures such as simple cluster counts as well as more complex characteristics showing cluster patterns and distributions were computed. The computed features include the mean and standard deviations of cluster size, within cluster dispersion, cluster extent and between cluster scatter [17]. We also computed the clustering indices such as Ball Hall, Banfeld-Raftery, LogSSRatio, Davies-Bouldin, Calinski-Harabasz, and C Index [17]. Moreover, we utilized proprietary graph features, which were constructed by analyzing the spatial relationships between the detected objects (PreciseDx, New York, NY).

### Statistical analysis

We trained and validated machine learning-derived image features using logistic regression to predict the disease status and stage and compared to expert assessments. There were 13 WSI per subject available for most cases, however, a few had more than 20 WSI. Each WSI was previously scored by three expert neuropathologists; the derivative scores were generated for each research subject [11, 13]. To generate one single feature vector for each subject, we used the maximum of each feature among all available slides, which performed better than the mean, median, and minimum.

We used a two-step process for selecting predictive features among all 60 engineered features and then trained a predictive model. To avoid overfitting, we split the subjects into train (80%) and test (20%) samples; then, used a logistic regression model with least absolute shrinkage and selection operator (LASSO) regularization to train a prediction model and select the most predictive features from the model. Since the number of subjects was limited (42 patients and 14 controls), the selected features would highly depend on the subjects in the training set. Hence, we repeated the random train/test split 1000 times and recorded the selected features and their importance weights for each split. The most frequently selected features with the largest importance weights were used to train and validate prediction models. We trained and validated 1000 Logistic Regression models using random train and test splits. We used an Elastic-Net penalty with *l1_ratio* = *0.01* (the Elastic-Net mixing parameter). Additional statistical analyses were performed using IBM SPSS Statistics version 26 and subscription for windows (IBM Corp., Armonk, NY, USA), which included Pearson and Spearman’s correlation analyses between AI features, expert scoring, and clinical characteristics; also Wilcoxon-Mann–Whitney rank-sum and Kruskal–Wallis analyses.

## Results

### Ground truth generation

First, to ensure reproducibility in our ground truth dataset used for training the neural network, we asked whether Lewy-type synucleinopathy (LTS) objects could be annotated in a consistent fashion and a minimal inter-observer variability bias. We performed a concordance study among five neuropathologists, using independent blinded assessments on 600 patches containing a range of LTS objects derived from a subset of the whole slide images (WSIs) of immunohistochemically stained submandibular gland biopsies from the Systemic Synuclein Sampling Study (S4) [1]. This balanced dataset was derived from prescreening and AI-detected false-positives from pilot studies (data not shown). Overall, we found a high degree of agreement between raters (5-way Fleiss’ kappa of 0.70, range 0.60–0.89 between pairs of raters; Additional file 1: Table S1). When ambiguous objects (“possible” LTS) were excluded, there was improvement in concordance of calls (Fleiss’ kappa of 0.73, range 0.84–0.90). This analysis prompted us to introduce a weighted ranking system allowing us delineate more concise operational definitions that included possible (1), probable (2), and definite (3) LTS ranked categories. These definitions were then deployed to generate the ground truth training dataset of annotated LTS objects from the training 283 WSI group from 56 research subjects, in which each LTS object was additionally ranked. WSI were then randomly divided into 80% for training and validation (i.e., model selection), with 20% reserved as a test set for performance evaluation (Additional file 1: Table S4). In total, we annotated 8450 positive foreground LTS objects and assigned each a confidence rank alongside a total of 35,066 background objects (Additional file 1: Table S5), which straightened the specificity of the detection.

### Convolutional neural network (CNN) training and validation

**Fig. 3:**
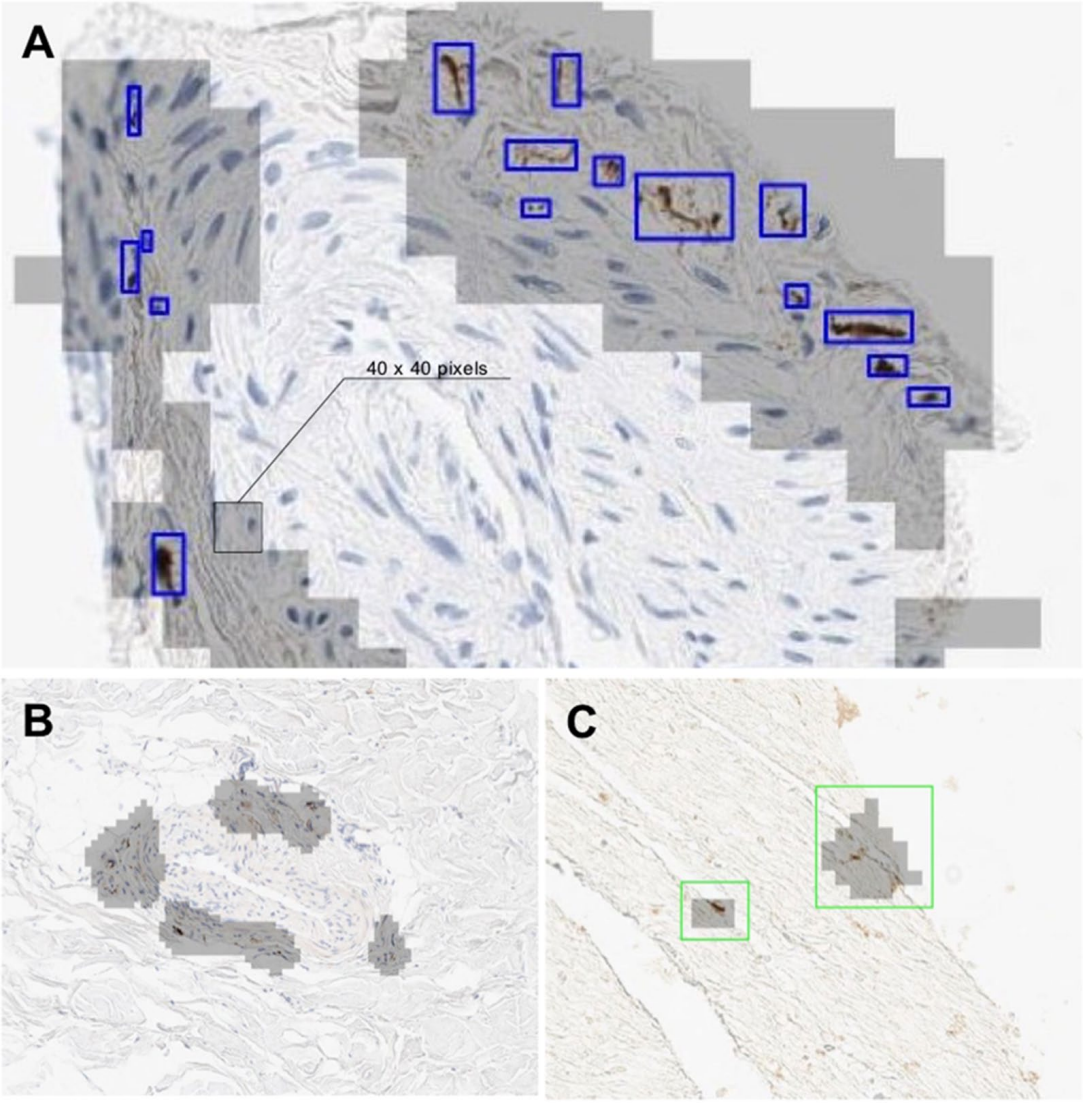
Examples of CNN deployment on SMG biopsy WSI. **A** An example of annotated objects (blue) and CNN inference (grey shading). A 40 × 40 pixel inference patch is shown. **B** An example of true positive CNN classification. **C** An example of false positive CNN classification. CNN, convolutional neural network; SMG, submandibular gland; LTS, Lewy-type synucleinopathy, WSI, whole slide image

For CNN training and validation, we conducted three rounds of annotation/training iterative cycles, triaging out false positives and triaging in false negatives, thus improving the ground truth and the accuracy of detection. The LTS detection time for a single WSI ranged from 10 to 40 min (averaging 18 min) with performance depending on the area of the tissue within the WSI. Examples of an annotated WSI with CNN inference, the 40 × 40-pixel overlapping inference patch indication, and examples of overlapping and true/false positive CNN detection inferences are shown (Fig. 3).

### Weighted CNN shows improved performance metrics

Each LTS object was assigned a confidence rank for weighted modeling to allow tunable penalties to improve network performance. This permitted us to train and compare weighted and unweighted CNNs (Table 2). We found that while both had excellent sensitivity/recall (0.99), the specificity was lower in the non-weighted version (0.92) compared to the weighted version (0.99). When the non-weighted and weighted network precision was compared there was a marked increase (0.41 vs. 0.81 respectively), which was also seen in F1 scores (0.59 vs. 0.89). Accuracy and area under the curve (AUC) receiver operating characteristics (ROC) were also higher in the weighted network. Thus, given the superior performance of the weighted CNN, it was used for further studies.

**Table 2:** Performance of non-weighted and weighted CNN LTS detectors

	Sensitivity/recall	Specificity	Precision	F1 score*	Accuracy	AUC**
Non-weighted	0.99	0.92	0.41	0.59	0.92	0.96
Weighted	0.99	0.99	0.81	0.89	0.99	0.99

*F1 score = 2 * Precision * Recall/(Precision + Recall)

**AUC: Area Under the Curve, Receiver Operating Characteristic

### Comparison of the CNN with expert scores

**Fig. 4:**
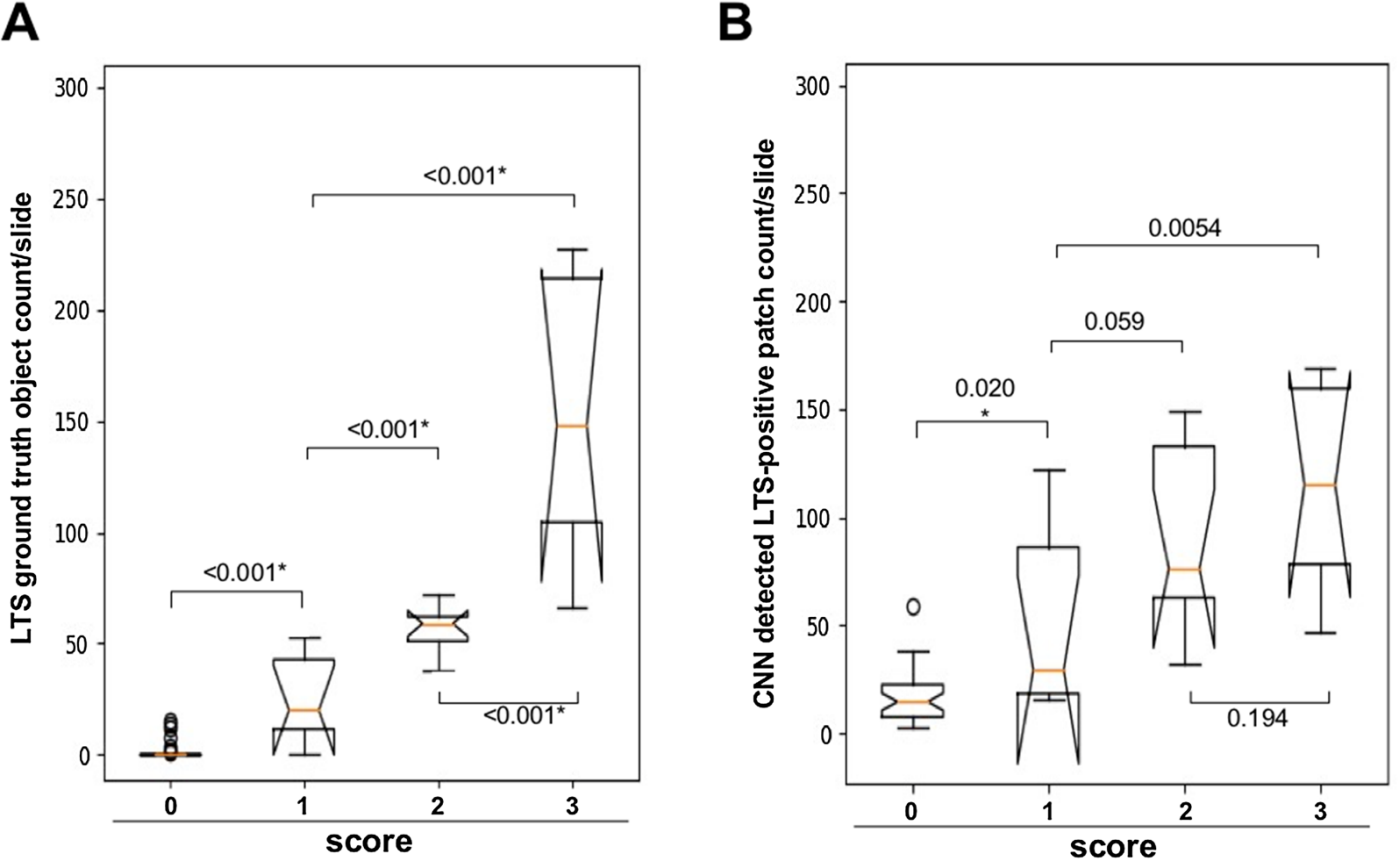
Comparison of ground truth annotations and CNN detection with expert scoring. **A** Expert annotation distribution boxplot in test WSI cohort (*n* = 56), Mann–Whitney two-tailed U test between score groups *p* values; Kruskal–Wallis H test of annotated LTS between expert score groups, and Spearman correlation between LTS burden and expert scores. **B** CNN, 40 × 40 patches positive for LTS distribution boxplot in test WSI cohort (*n* = 56), Mann–Whitney two-tailed U test between score groups *p* values; Kruskal–Wallis H test of 40 × 40 patches between expert score groups, and Spearman correlation between 40 × 40 patches positive for LTS burden and a score in test cohort. Scoring was performed as follows: each WSI was classified as positive or negative for α-synuclein pathology and assigned an LTS score ranging from 0 to 3 (Fig. 1), where 0 refers to being negative for α-synuclein, and scores 1–3 refer to scoring density of sparse (1) moderate (2), and frequent (3). *CNN* convolutional neural network, *SMG* submandibular gland, *LTS* Lewy-type synucleinopathy

**Fig. 5:**
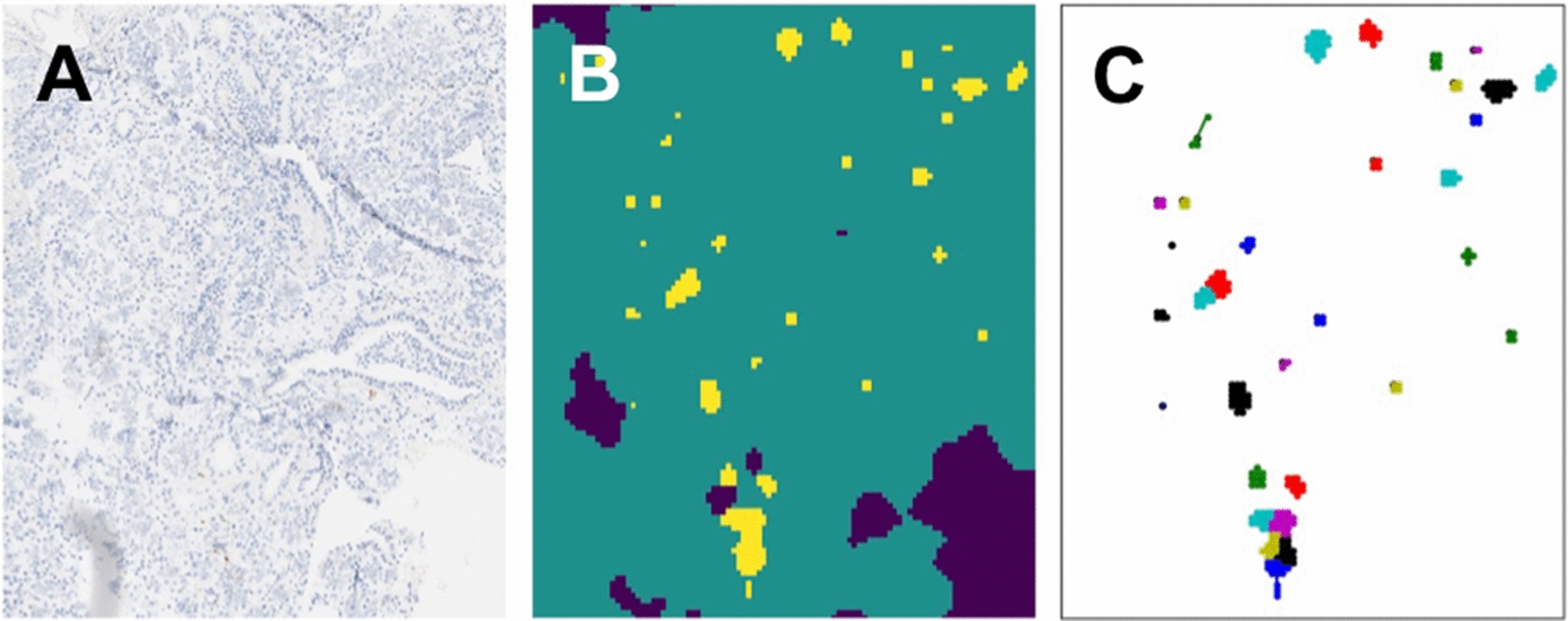
Examples of CNN deployment on SMG biopsy WSI. **A** An Example of a WSI. **B**, **C** LTS patch detection and features generation (e.g., patches clustering, in color). *CNN* convolutional neural network, *SMG* submandibular gland, *LTS* Lewy-type synucleinopathy

We next compared annotation ground truth and the CNN LTS object detection with the expert scoring performed in our previous study [13]. Ground truth annotation follows the same trend and expert scoring with good distinction between scored groups (Fig. 4A). These expert scores represent the subjective assessment from the neuropathologist evaluators, integrating rater confidence of object identity alongside other features such as cellular context. In the set of WSI used for network training that were scored as negative, on average 1.6 LTS objects were identified and included in our annotations, and 17 CNN detected LTS-positive patches (Fig. 4B, Additional file 1: Table S6). Overall, we found a highly significant correlation between expert scores and annotated ground truth as well as the CNN LTS detection (4.3 × 10^−69^, Rho = 0.82 and 9.9 × 10^−12^, 0.76 respectively). There was a highly significant difference in ground truth annotated objects count and CNN-detected LTS-positive patches count between expert scored groups (*p* = 4.7 × 10^−7^, Kruskal–Wallis). The strong correlation between our ground truth object annotations and the expert LTS burden scores gave us further confidence in the quality of our ground truth. The strong correlation between both these measures and the CNN scores gave us further confidence in our network (Fig. 5).

### CNN-derived features improve prediction of clinical PD status

In order to comprehensively evaluate the LTS CNN, it was necessary to test it on a set of naïve images. We used WSI (*n* = 1230) of additional sections from the SMG tissue blocks also stained using α-synuclein immunohistochemistry derived from the cohort of PD patients (n = 42) and controls (n = 14). We used our CNN-derived LTS-positive patches to generate a set of features that could be used for correlation analyses with clinical outcomes and neuropathological assessments, and improve the predictive power of our model. We selected 13 high-ranking features for PD status prediction out of the initial set of 60 AI features using a logistic regression model and a least absolute shrinkage and selection operator (LASSO) shrinkage method for pruning. The expert slide level score specificity prediction of disease status is the same as reported previously [13] and higher than AI specificity in our series (Table 3). The logistic regression analysis showed that the prediction of PD status was slightly more accurate with AI features compared to the expert scoring and its derivatives. The mean and standard deviation of AI features performance metrics in predicting the PD status are compared to those of the models based on the expert assessment scores and derivative features (Table 3).

**Table 3:** Prediction of Parkinson disease status, logistic regression, *n* = 56 (14 controls and 42 PD), Mean ± SD

	Sensitivity/recall	Specificity	Precision	F1 score*	Accuracy
13 AI features altogether	0.71 ± 0.16	0.65 ± 0.30	0.86 ± 0.13	0.76 ± 0.12	0.69 ± 0.13
Expert score and its derivatives	0.59 ± 0.16	0.88 ± 0.24	0.94 ± 0.13	0.71 ± 0.13	0.66 ± 0.13
Expert score	0.54 ± 0.16	0.93** ± 0.17	0.96 ± 0.10	0.68 ± 0.14	0.64 ± 0.13
Expert score derivatives	0.60 ± 0.16	0.89 ± 0.23	0.94 ± 0.11	0.72 ± 0.13	0.67 ± 0.13

*F1 score, harmonic mean is calculated as 2 * Precision * Recall/(Precision + Recall)

**Expert score specificity is the same as reported previously [6]

### AI-derived object features improve accuracy of PD stage prediction

In order to improve PD staging prediction modeling, we selected 5 high-ranking features for PD stage out of the initial set of 60 AI features using the same logistic regression model and a LASSO shrinkage method for pruning (Additional file 1: Table S3). The accuracy of prediction of PD stage also increased with the use of AI compared to the models using expert scoring. This improvement was more prominent while assessing the difference between PD stages only (0.64 *v.* 0.59), whereas the difference in accuracy is lower (0.48 *v*. 0.45) when controls were taken into the analysis. Accuracy is based on both sensitivity and specificity, however, while AI had higher sensitivity, expert scoring has higher specificity, when distinction of PD from controls is the criterion (Table 4). To determine how well the selected 15 AI features, which are predictive of PD status and stage, correlate with expert scoring we applied Spearman’s correlation analysis. It showed that 14 out 15 selected AI features were in significant (*p* < 0.001) correlation with expert scoring on individual slides and 13 out of 15 were in significant (*p* < 0.001) correlation a summarized synuclein score per patient (Table 5).

**Table 4:** Accuracy of prediction of clinical Parkinson disease stage by the weighted CNN*

	Stages 0–3 *(n* = 56)	Stages 1–3 (*n* = 42)
Five AI features	0.48 ± 0.14	0.64 ± 0.16
Expert scoring and its derivatives	0.45 ± 0.14	0.59 ± 0.17

*Mean ± SD

Parkinson disease stages: early [1], moderate [2], advanced [3], and controls [0]

**Table 5:** Selected highest ranking AI features correlations with expert scoring, Spearman's rank correlation coefficient *Rho*

	Per slide score, *n* = 1230		Per subject score, *n* = 56	
	*Rho*	*p* Value	*Rho*	*p* Value
LTS cluster size SD	**0.75**	**1.20E**−**225**	**0.84**	**4.83E**−**16**
PreciseDx Graph Feature18	**0.28**	**8.78E**−**24**	**0.70**	**1.54E**−**09**
StDev of Hematoxylin Channel	**0.61**	**1.82E**−**128**	**0.65**	**7.59E**−**08**
PreciseDx Graph Feature5	**0.77**	**4.18E**−**237**	**0.75**	**2.85E**−**11**
PreciseDx Graph Feature29	**0.69**	**1.44E**−**171**	**0.81**	**3.06E**−**14**
PreciseDx Graph Feature11	**0.60**	**4.50E**−**122**	0.15	0.269
Davies-Bouldin Index	**0.56**	**8.90E**−**101**	**0.53**	**3.23E**−**05**
PreciseDx Graph Feature13	**0.71**	**7.91E**−**189**	**0.79**	**5.02E**−**13**
C Index	**0.65**	**1.54E**−**150**	0.28	0.035
PreciseDx Graph Feature28	**0.71**	**1.06E**−**185**	**0.82**	**1.50E**−**14**
PreciseDx Graph Feature14	**0.36**	**3.91E**−**38**	**0.69**	**4.95E**−**09**
PreciseDx Graph Feature10	0.04	0.159	− 0.22	0.106
Calinski-Harabasz index	**0.66**	**3.58E**−**156**	**0.61**	**6.66E**−**07**
PreciseDx Graph Feature3	**0.75**	**1.20E**−**220**	**0.80**	**8.88E**−**14**
PreciseDx Graph Feature27	**0.73**	**6.00E**−**201**	**0.78**	**1.54E**−**12**

Statistically significant values are shown in bold

*LTS* Lewy type synucleinopathy

### AI-derived object features distinguish between PD status and PD stages

To confirm that the 13 high-ranking AI features, derived with mathematical modeling, are capable of reliably distinguishing between PD and controls, we applied Wilcoxon–Mann–Whitney analysis, in which 8 of 13 selected features have shown statistically significant differences between PD patients and controls in selected ranked measures. Of note, all the most significant AI features correlates are reflecting the LTS clustering characteristics. Other features, unrelated to LTS objects clustering, that show the difference between PD and controls, are reflecting the LTS burden: the standard deviation of the DAB channel and the standard deviation of the hematoxylin channel (Table 6). We also applied the Kruskal–Wallis analysis to verify that those selected features are capable of reliably distinguishing among PD stages (early [1], moderate [2], and advanced [3]), in which 4 of 5 selected features, all reflecting the LTS clustering characteristics, have shown statistically significant differences among the PD stages (Table 6).

**Table 6:** Selected highest ranking AI feature differences between Parkinson disease and controls (Mann–Whitney U test), and between stages (Kruskal–Wallis test)

AI features	Mann–Whitney U test	
	U	*p* Value
PreciseDx Graph Feature27	**143.0**	**0.001**
LTS cluster size StDev	**122.0**	**0.001**
PreciseDx Graph Feature3	**138.0**	**0.002**
PreciseDx Graph9	**170.0**	**0.009**
StDev of Hematoxylin Channel	**175.5**	**0.025**
PreciseDx Graph Feature14	**210.0**	**0.027**
Calinski-Harabasz index	**179.5**	**0.030**
PreciseDx Graph Feature13	**201.5**	**0.047**
PreciseDx Graph Feature28	208.5	0.056
Davies-Bouldin Index	204.0	0.088
PreciseDx Graph Feature11	249.0	0.338
PreciseDx Graph Feature10	268.0	0.419
C index	266.5	0.601
LTS pixel fraction	**168.0**	**0.017**
StDev of DAB channel (brown)	**173.5**	**0.023**
Number of LTS patches	**153.0**	**0.008**

	Kruskal–Wallis test	
	Kruskal–Wallis H	*p* Value
PreciseDx Graph Feature4	**11.6**	**0.003**
PreciseDx Graph Feature17	**10.9**	**0.004**
PreciseDx Graph Feature3	**7.5**	**0.024**
LTS cluster size StDev	**7.0**	**0.03**
PreciseDx Graph Feature27	4.8	0.089

Statistically significant values are shown in bold

Controls, n = 14; PD patients, n = 42; PD early stage patients, n = 15; PD moderate stage patients, n = 13; PD advanced stage patients, n = 14

*LTS* Lewy type synucleinopathy

### AI-derived features correlation with UPDRS, CSF α-synuclein, and neuroimaging markers

We also explored the relationship between the set of 15 selected AI features and clinical and biomarker measures of PD disease severity namely MDS-UPDRS total and part III scores (where higher values indicate more severe disease), CSF total α-synuclein, and dopamine transporter binding in striatum measured with SPECT (DAT-SBR; where lower values indicate lower (worse) DAT binding). Pearson correlation showed statistically significant, although moderate, inverse relationships between some AI features and median bilateral striatal DAT-SBR in PD patients. Lower correlations were observed between AI features and MDS-UPDRS scores and CSF α-synuclein biochemical values (Table 7).

**Table 7:** Selected highest ranking AI features correlation with UPDRS score, CSF biochemistry, and dopamine transporter SPECT, Pearson *r*

AI features	MDS-UPDRS Part III, *n* = *55*		MDS-UPDRS total, *n* = 55		DAT-SBR*, *n* = 56		CSF Synuclein, *n* = 53	
	*r*	*p* Value	*r*	*p* Value	*r*	*p* Value	*r*	*p* Value
LTS cluster size StDev	**0.35**	0.010	**0.33**	**0.014**	− **0.39**	**0.003**	0.08	0.556
PreciseDx Graph Feature18	**0.39**	0.003	**0.37**	**0.005**	− **0.50**	**8.21E**−**05**	0.14	0.303
StDev of Hematoxylin Channel	0.16	0.259	0.14	0.312	− **0.29**	**0.033**	0.13	0.369
PreciseDx Graph Feature5	**0.35**	0.009	**0.29**	**0.031**	− **0.43**	**0.001**	**0.05**	0.728
PreciseDx Graph Feature29	**0.38**	0.004	**0.36**	**0.007**	− **0.43**	**0.001**	0.09	0.504
PreciseDx Graph Feature11	− 0.07	0.609	− 0.13	0.361	0.01	0.928	0.19	0.173
Davies-Bouldin Index	0.09	0.510	0.08	0.547	− **0.30**	0.025	0.25	0.066
PreciseDx Graph Feature13	**0.27**	0.044	0.23	0.086	− **0.35**	**0.009**	0.11	0.454
C Index	− 0.16	0.259	− 0.14	0.318	0.06	0.671	0.07	0.616
PreciseDx Graph Feature28	**0.31**	0.022	**0.33**	**0.014**	− **0.41**	**0.002**	0.12	0.408
PreciseDx Graph Feature14	**0.32**	0.016	**0.30**	**0.026**	− **0.35**	**0.007**	− 0.14	0.333
PreciseDx Graph Feature10	− 0.11	0.443	− 0.11	0.444	0.11	0.423	**0.27**	0.049
Calinski-Harabasz index	**0.31**	0.021	**0.27**	0.044	− **0.32**	**0.017**	0.07	0.616
PreciseDx Graph Feature3	**0.44**	0.001	**0.40**	**0.003**	− **0.50**	**7.48E**−**05**	0.07	0.625
PreciseDx Graph Feature27	**0.36**	0.006	**0.34**	0.011	− **0.50**	**8.11E**−**05**	0.07	0.629

Statistically significant values are shown in bold

*LTS* Lewy type synucleinopathy, *MDS-UPDRS* Movement Disorders Society Unified Parkinson Disease Rating Scale

*DAT-SBR, dopamine transporter mean striatum specific binding ratio

## Discussion

It is well established that while Parkinson’s disease pathologically manifests in the CNS with aggregation of α-synuclein as Lewy bodies and Lewy neurites, this pathology can also be found in the peripheral nervous system. Peripheral Lewy-type synucleinopathy objects (LTS) are present in PD, suggesting its utility as a diagnostic and prognostic biomarker [11]. Previous studies have shown that the expert neuropathologist semi-quantitative scoring of immunohistochemically stained WSI from PD patients, based on LTS density in the submandibular gland has high specificity in diagnosing PD, but suboptimal sensitivity. In this study, we present a novel machine learning-based method using a total LTS burden as well as clustering characteristics reflecting LTS density and distribution on digitized IHC-stained preparations. Our study demonstrates that deep machine learning represents a feasible way to augment routine histological examination, and trained neural networks could be deployed in detecting the peripheral LTS and improving accuracy for further confirmatory assessment by a neuropathologist.

A critical step for training a neural network is to minimize the inter-observer variation to develop the most robust ground truth. We designed an inter-rater reliability study and conducted consensus conferences among raters to improve the operational definitions for ground truth objects. We have achieved a high degree of agreement among raters and went through iterative rounds of discussions about ambiguous and challenging objects. We trained a CNN to identify image patches that contain LTS objects. We also conducted three rounds of annotation/training iterative cycles triaging out false positive and triaging in a false negative, which resulted in the high-quality ground truth and further translated into high accuracy of LTS detection (Table 2). Since there were many LTS objects on each slide, especially those with higher burden, comprehensive annotation of all individual LTS objects was not practical. Also, there were many small objects densely packed with larger objects that made it very difficult to localize and annotate them. Hence, rather than using object localization methods such as region-based CNN (RCNN) or single shot multibox detector (SSD), we opted to use CNN to determine whether an image patch contains any (one or multiple) LTS object. Using the confidence score of the annotated objects as error weights in the loss function (when training the CNN classifier) also helped us to increase the sensitivity of the detector in identifying definite and probable LTS objects.

We showed that AI-based LTS detection and distribution characteristics correlate well with expert scoring performed in the previous study, but AI may offer greater speed and higher sensitivity. It currently takes our LTS classifier an average of 18 min to computationally screen an entire WSI and highlight the ROI with a high probability of LTS. Fully automatic detection of LTS at this performance level will enable large-scale screening of WSI for further confirmation by a human expert neuropathologist. This illustrates the feasibility of applying this approach to large datasets and paves the road to further adopting this approach in clinical practice. We also demonstrated that AI has higher accuracy, sensitivity, and F1 score in predicting PD status than human expert scoring; as well as higher overall accuracy in predicting PD progression. Even though the specificity of an AI in predicting PD status was lower than expert neuropathologist scoring, the current version of trained AI shows to be promising in the ability to reproducibly identify high probability LTS foci on a digital slide.

We showed that LTS cluster size characteristics as well as the graph features reflecting the spatial relationships between the detected objects were the highest-ranking features that correlated the best with the expert score per slide and per patient (Table 5). The highest-ranking features to differentiate the controls and PD and to detect the difference among the PD stages are all clustering characteristics, whether they are well-known (e.g., Calinski-Harabasz index or LTS cluster size standard deviation) or the proprietary graph features newly developed by PreciseDx (Table 6). AI features, determined using mathematical modeling, can be potentially useful to clinical-pathological-radiological correlations, exemplified with the statistically significant, although moderate, inverse relationships between selected AI features and median bilateral striatal DAT-SBR in PD patients (Table 7).

In our study, we showed that with our initial training of the CNN detector, it can achieve a similar outcome compared to the expert performance. However, the AI approach is shown to be more reliable, consistent, and cost-efficient compared to the old-fashioned way. The expert could be subjected to inter and intra-observer variation while AI is consistent and able to offer the most accurate result possible. To train a neuropathologist requires a considerable investment of both money and time. In addition to the extensive training, it could still take an expert a substantial time to fully annotate an entire WSI. While AI can annotate an entire WSI in mere minutes, and with a larger sample set AI can continue perfecting itself.

Expert scoring and AI-based LTS burden and clustering characteristics correlate well, but AI may have some advantages. On the currently limited dataset, AI has shown to have (1) high correlation with expert scoring; (2) higher accuracy, sensitivity, and F1 score in predicting PD status than expert scoring; (3) higher accuracy in predicting PD progression than expert scoring, albeit with less specificity. Overall, we found that LTS clustering characteristics are a better predictor of the outcome than crude LTS burden. Our trained weighted InceptionV4 neural network is promising in its ability to reproducibly identify high probability LTS foci on a digital slide. Together with the subsequent mathematical modeling algorithms reflecting LTS clustering characteristics offer a useful tool in assessment and screening of histological slides for total α-synuclein burden and for prediction of PD status and stage of progression and for further utilization in practical clinical applications.

This study had several notable limitations. Of note, we had a relatively small dataset. In the future, larger datasets may allow us to develop more sensitive and specific tests. Another limitation is that we focused our effort on SMG biopsies stained with the 5C12 anti-α-synuclein antisera by immunohistochemistry. It is unclear if this approach would be valid in other tissues or other antisera, which is a critical question as interest in skin biopsies for the diagnosis of PD is growing and expanding our analysis to skin and colon biopsies would be of value. Also, it is unclear whether other α-synuclein antibodies would give similar results. Potentially, we further plan to expand the subject cohort for better neural network training. Because of the nature of the study, there was no autopsy material available for definitive neuropathological diagnosis and clinical-pathological correlations. Expanding the project to include autopsy subjects will allow us to perform AI-clinical-pathological correlations.

In conclusion, we demonstrated the promise of AI in aiding an expert in making an antemortem diagnosis of definite PD, and potentially other synucleinopathies, such as dementia with Lewy bodies and multiple system atrophy. We will continue broaden and improve our research protocol and approach to the application of AI in neuropathology. The application of AI in clinical pathology is limitless, the quantitative data uncovered by AI networks will not only augment the currently used qualitative and semi-quantitative approach in the assessment of the pathognomonic features, but also be used for correlative and association analyses with clinical, radiological, genetic, and biochemical data.

## Supplementary Information

**Additional file 1:** Supplementary tables.

## Data Availability

De-identified clinical data collected for this study will be available at https://braincommons.org. Data requestors will need to sign a data use agreement via the BRAIN Commons website (https://braincommons.org) to gain access. Additional computational data will be accessible on obtaining permission on GitHub: https://github.com/PreciseDx/S4-MJFF.

## Abbreviations

AI: Artificial intelligence
CSF: Cerebrospinal fluid
DAT: Dopamine transporter
DAB: Diaminobenzidine
IHC: Immunohistochemistry
LTS: Lewy-type synucleinopathy
MDS-UPDRS: Movement Disorders Society Unified Parkinson Disease Rating Scale
MDS-UPDRS-III: Movement Disorders Society Unified Parkinson’s Disease Rating Scale part III
PD: Parkinson disease
S4: Systemic Synuclein Sampling Study
SBR: Dopamine transporter mean striatum specific binding ratio
SPECT: Single-photon emission computer tomography
SMG: Submandibular gland
